# PMTCT of HIV-1 in Burkina Faso: evaluation of residual vertical transmission by PCR, molecular characterization of subtypes and determination of antiretroviral drugs resistance

**DOI:** 10.1186/1471-2334-14-S2-P59

**Published:** 2014-05-23

**Authors:** T Sagna, C Bisseye, TS Kagone, FW Djigma, D Ouermi, MTA Zeba, VJT Bazié, Z Douamba, R Moret, V Pietra, A Koama, S Pignatelli, C Gnoula, JD Sia, JB Nikiema, J Simpore

**Affiliations:** 1 Pietro Annigoni Biomolecular Research Center (CERBA), Ouagadougou, Burkina Faso

## Aim

Vertical HIV transmission is a public health problem in Burkina Faso. This study on the prevention of mother to child HIV-1 transmission had the objectives to: i) determine the residual rate of vertical HIV transmission by PCR ii) Detect of HIV antiretroviral drugs resistance among mother-infant pairs and iii) Identify sub-types and Circulating recombinant form (CRF) in Burkina Faso.

## Materials and methods

In this study, 3,215 samples of pregnant women were analyzed using rapid tests "Determine HIV1/2". Vertical transmission was estimated by PCR in newborns from those who were HIV positive. HIV-1 Resistance to ARVs, subtypes and CRFs were determined through ViroSeq kit using the ABI PRISM 3130 sequencer.

## Results

In this study, 12.26% (394/3215) of the pregnant women were diagnosed HIV-positive, 0.52% (2/388) overall residual rate of transmission was identified with rates of 1.75% (2/114) among mothers under prophylaxis and 0.00% (0/274) for those under HAART. Genetic mutations have also been isolated that induce resistance to ARVs like M184V, Y115F, K103N, Y181C, v179E, G190A, etc. And there were subtypes and circulating recombinant forms (CRF) of HIV-1 such as: CRF06_CPX (58.8%), CRF02_AG (35.3%) and subtype G (5.9%).

## Conclusion

Antiretroviral drugs reduce the residual rate of HIV vertical transmission. However, they cause mutations that induce resistance of HIV to antiretroviral therapy. Resistance to ARV therefore requires a permanent dialogue between clinicians, prescribers, pharmacists, and the creation of a network of monitoring and surveillance of drug resistance in Burkina Faso.

